# Monitoring the Efficiency of *Rhazya stricta* L. Plants in Phytoremediation of Heavy Metal-Contaminated Soil

**DOI:** 10.3390/plants9091057

**Published:** 2020-08-19

**Authors:** Ehab Azab, Ahmad K. Hegazy

**Affiliations:** 1 Biotechnology Department, Faculty of Science, Taif University, Taif 21974, Saudi Arabia; 2 Botany and Microbiology Department, Faculty of Science, Zagazig University, Zagazig 44519, Sharkia, Egypt; 3 Botany and Microbiology Department, Faculty of Science, Cairo University, Giza 12613, Egypt; hegazy@sci.cu.edu.eg

**Keywords:** phytoremediation, *Rhazya stricta*, heavy metals, contaminated soil, arid lands

## Abstract

Heavy metal-contaminated soil constitutes many environmental concerns. The toxic nature of heavy metals poses serious threats to human health and the ecosystem. Decontamination of the polluted soil by phytoremediation is of fundamental importance. Vegetation is an appealing and cost-effective green technology for the large-scale phytoremediation of polluted soils. In this paper, a greenhouse experiment was carried out to test the potential of *Rhazya stricta* as a heavy metal phytoremediator in polluted soil. Plants were grown for three months in pots filled with soils treated with the heavy metals Cd, Pb, Cu, and Zn at rates of 10, 50, and 100 mg/kg. The bioaccumulation factor (BCF) and translocation factor (TF) were calculated to detect the ability of *R. stricta* to accumulate and transfer heavy metals from soil to plant organs. The results showed that under increasing levels of soil pollution, the bioconcentration of Cd and Zn heavy metals showed the highest values in plant roots followed by leaves, whereas in the case of Pb and Cu, roots showed the highest values followed by stems. Heavy metals accumulation was higher in roots than in stems and leaves. The BCF of Zn reached the highest values in roots and stems for 10 mg/kg soil treatment, followed by the BCFs of Cd, Cu, and Pb. The TF for the different heavy metal pollutants’ concentrations was less than unity, suggesting that the plants remediate pollutants by phytostabilization. The TF values ranged from higher to lower were in the order Zn > Cu > Cd > Pb. The rapid growth of *R. stricta* and its tolerance of heavy metals, as well as its ability to absorb and accumulate metals within the plant, recommends its use in the phytoremediation of slightly polluted soils in arid lands by limiting the heavy metals transport.

## 1. Introduction

Environmental contamination by heavy metals is becoming a critical problem of global concern. The toxic metals may remain in the environment for a long period and can eventually accumulate to levels that could cause major threats to human health [1].

Heavy metals are considered to be non-degradable inorganic pollutants, which are released to the environment from both human and natural activities, causing ecosystem disturbance [2]. Both volcanoes and parent material weathering are among the natural sources of heavy metal pollution [3]. Human activities, including mining, the use of fertilizers, pesticides and sludge as well as intensive asphaltic road construction across the natural landscape, are the main sources of heavy metal contamination of non-polluted soils, which in turn affects the food chain, leading to environmental and human health risks [4,5,6,7]. According to their importance to living organisms, heavy metals are classified into essential and nonessential metals. Among the essential metals are Fe, Ni, Cu, Mn, and Zn, which have physiological functions in plant growth and development [8]. Pb, Cd, As, and Hg are nonessential because they do not have significant roles in the physiological functions of plants [9]. Although phytoextraction requires a long time to clean up heavy metals from contaminated soil, its cost-effectiveness and low environmental impact outweigh the time factor [9,10].

Many biological methods have been designed to improve the phytoremediation mechanisms utilized in reducing, removing or extracting heavy metals from the contaminated zones. Decontamination of the polluted soil by phytoremediation is considered as an effective method using plants to remediate the hazardous waste sites [11].

Phytoremediation is a green cost-effective eco-approach used in cleaning up soil and water from the chemical contaminations. As an essential method utilized in reducing the environmental pollution, an interest in phytoremediation has been growing rapidly in recent years [10,12,13,14,15,16,17]. As an emerging and sustainable technology that is cost-effective, solar-driven, passive and aesthetically acceptable, phytoremediation has become a sustainable technology for contaminant removal that can be used in different habitat types [16]. Chemical or physical techniques such as chemical washing and incineration are costly remediation methods that affect soil properties [4].

Identifying the most effective phytoremediator plant species that resist or accumulate chemical pollutants in soil, water, and air will support the use of green technology for the reclamation of polluted ecosystems in different zonobiomes.

Natural vegetation is playing an essential ecological role in the recovery of disturbed and contaminated land [18,19,20,21]. Recently, the use of vegetation has represented an innovative green technique for the in situ and ex situ phytoremediation of polluted soils. The vegetation accumulates heavy metals and other chemical pollutants and thereby reduces their risk to the environment [5,22,23].

In some plant communities, *Rhazya stricta* Decne is considered a keystone species that determines the general appearance of the plant community and maintains local biodiversity through its impact on the abundance and type of other species as a critical component of the food web in many locations [24]. Populations of *R. stricta* shape the general appearance of the plant community by accumulating sand mounds that form mound-field landscapes. These create shelter for burrowing animals and are considered nutrient islands that sustain many different plants and animals in the ecosystem. As ecosystem engineers, keystone species can create and modify as well as maintain the landscape around them. They influence the occurrence of other organisms and help define the overall biodiversity of their habitat [25].

*R. stricta* is an efficacious medicinal plant used in herbal drugs to cure abundant ailments [26]. In spite of the medicinal value of *R. stricta* Decne, medicinal wild plants in arid lands are not collected at a large scale through industrialization because of their low biomass productivity but they are cultivated in agricultural lands for commercial use. Studying the heavy metal accumulation potential of *R. stricta* may result in a recommendation not to cultivate the species for medicinal purposes in heavy metal-polluted areas or areas watered with polluted water.

Phytoremediation of heavy metal-polluted soils using *R. stricta* plants, and their ability to absorb and accumulate heavy metals, have been investigated in various research topics. It was revealed that the accumulation factor for copper, cadmium, lead, and zinc was higher in roots of *R. stricta* compared with stems or leaves [27]. Moreover, *R. stricta* plants were suggested as an indicator for soil contamination, especially with Cd and Pb heavy metals [28]. *R. stricta* plants were also utilized to remove arsenic (As) from contaminated aqueous solutions [29]. Furthermore, *R. stricta* plant species were considered as resistant plants as these plants contain in their tissues amounts of heavy metals higher than those considered toxic for plants [30].

For some native plants such as *R. stricta*, *A. Mexicana*, *C. colocynthis*, *C. italic*, *P. australis*, *C. laevigatus*, and *C. procera,* the bioaccumulation factor values of Cd, Cu, Ni, and Zn were higher than the values of Cr, Co, Fe, and Pb heavy metals [31]. Further, some wild plants such as *R. stricta*, *L. shawii*, *M. parviflora*, and *P. australis* plants were effective in phytoextraction of Cd and Pb heavy metals [32]. Some plant species such as *R. stricta*, *E. cornigera*, and *C. colocynthis* were suggested as bio-indicators and accumulators for contaminated soil [33]. In addition, some medicinal plants such as *R. stricta*, *C. spinosa*, *P. harmala*, and *T. articulata* were recommended to accumulate Fe, Ni, Mn, Zn, Cu, Cd, Cr, and Pb heavy metals at different concentrations [34].

Further intensive studies on *Rhazya stricta* plants recommended their use in the phytoremediation of toxic heavy metals in the contaminated natural areas of many countries where they grow. The major goal of this study is to investigate the efficiency of *Rhazya stricta* L. plants in the phytoremediation of heavy metal-contaminated soil. The study was conducted as a pot experiment in an open greenhouse by including the usually accompanied contaminant heavy metals copper, zinc, cadmium, and lead.

## 2. Results

### 2.1. Growth Rate and Resource Allocation

**Figure 1:**
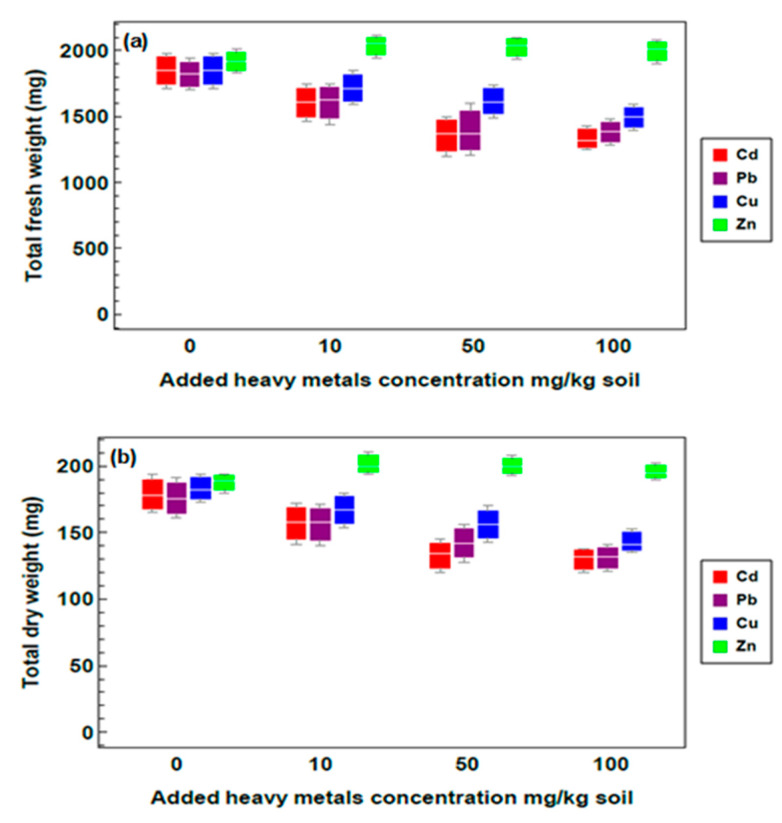
Growth rate of *R. stricta* plants exposed to different heavy metal treatments. (**a**) Fresh weight, and (**b**) dry weight of *R. stricta* plants samples under different treatments (means ± standard errors (*n* = 5) for different heavy metal treatments).

The growth rate of *R. stricta* plants under different heavy metal treatments is illustrated in Figure 1. The obtained results showed that there is no significant difference at 10 and 50 mg/kg heavy metals concentration for both fresh and dry weight. For 100 mg/kg heavy metals concentration, a slight decrease in biomass compared with the control group was remarked (Figure 1a,b).

**Figure 2:**
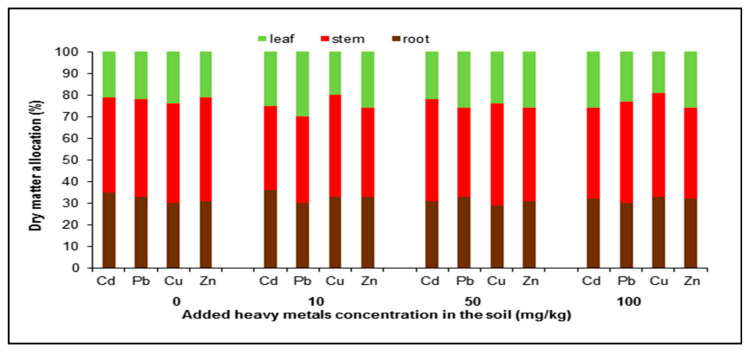
Dry matter allocation (%) of *R. stricta* plants under different heavy metal treatments across different plant organs. Soil treated with different heavy metals (Cd, Pb, Cu, and Zn) concentrations 10, 50 and 100 mg/kg soil in an open greenhouse.

Dry matter allocation of *R. stricta* plants under different heavy metal treatments across different plant organs was introduced in Figure 2. The results in this figure indicated that, the percentage of root dry weight ranged from 30% to 35% for control, while for 100 mg/kg soil heavy metal treatments it ranged from 30% to 33%. This indicates that there is no significant change in the root dry weight even at 100 mg/kg heavy metal treatments. Since the plants were grown in the greenhouse, non-significant root growth was observed. The same pattern was also detected for stem and leaf as shown in Figure 2. Therefore, dry matter allocation in *R. stricta* plant organs was not affected by the applied levels of concentration of different heavy metals.

### 2.2. Concentration of Heavy Metals

**Figure 3:**
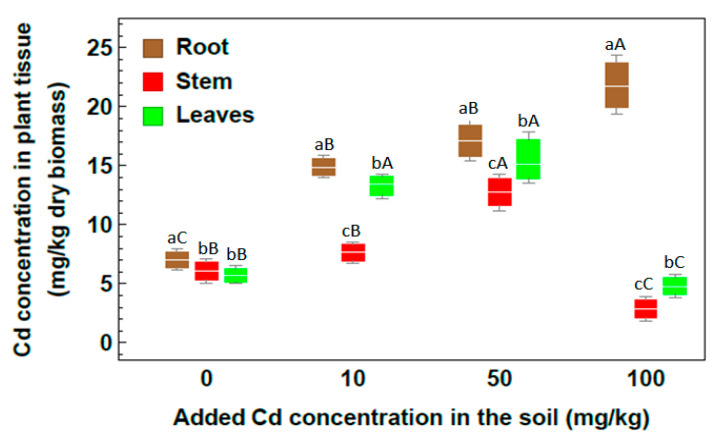
Concentration of Cd in plant organs of *Rhazya stricta* grown in soil treated with 10, 50 and 100 mg Cd/kg soil in an open greenhouse. Different lowercase letters indicate significant differences between plant parts for each soil metal concentration, and different capital letters indicate a significant difference between soil metal concentrations for each plant part (*p* < 0.05).

Heavy metals uptake by *Rhazya stricta* plants through the phytoremediation mechanism was observed as a clear increase in heavy metals concentration within different plant organs. For Cd heavy metal (Figure 3), the highest concentration value was 21.87 mg/kg in plant roots under the 100 mg/kg Cd soil treatment, compared with the concentration in the roots of the control, at 7.01 mg/kg. The highest concentration for leaves and stem were recorded in the 50 mg/kg soil treatment since they were 15.5 and 12.76 mg/kg, respectively. Accumulated Cd in stem at 100 mg/kg treatment was much lower than in other treatments with a value of 2.87 mg/kg. At 100 mg/kg Cd concentration, a significant increase was remarked in root, whereas a significant decrease was remarked for stem and leaves.

**Figure 4:**
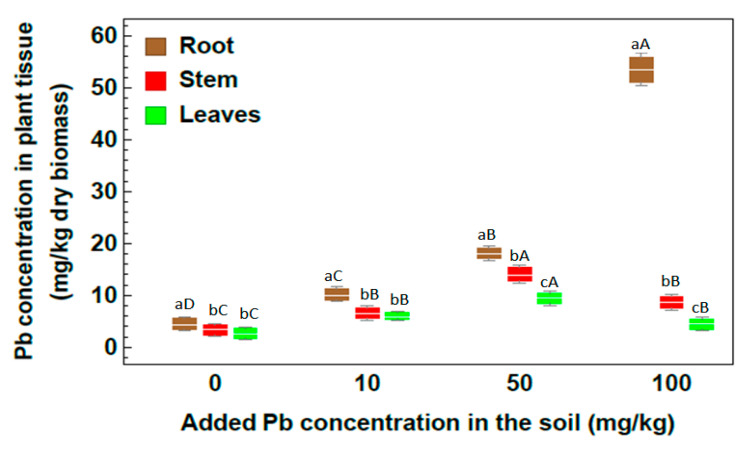
Concentration of Pb in plant organs of *Rhazya stricta* grown in soil treated with 10, 50 and 100 mg Pb/kg soil in an open greenhouse. Different lowercase letters indicate significant differences between plant parts for each soil metal concentration, and different capital letters indicate a significant difference between soil metal concentrations for each plant part (*p* < 0.05).

The Pb concentration in the *R. stricta* organs was higher in all Pb soil treatments than in the control (Figure 4). The Pb concentration in the roots was significantly higher than that in the stem and leaves. The maximum Pb concentration was 56.20 mg/kg in the roots of plants grown in soils treated with Pb at 100 mg/kg. The Pb concentration in the stem was higher than that in the leaves under all Pb treatments. The results illustrated that within 50 mg/kg Pb concentration, *R. stricta* plants showed high efficiency for absorbing Pb heavy metal and transferring it to the aerial parts, whereas in 100 mg/kg Pb concentration, the root of *R. stricta* showed the highest absorption rate and failed to transfer Pb heavy metal to the aerial parts.

**Figure 5:**
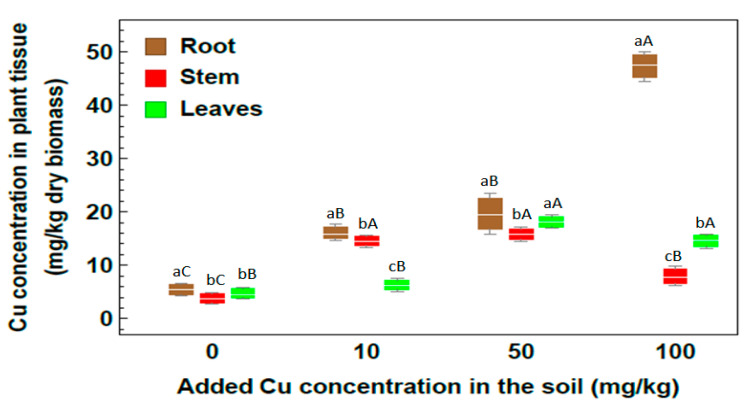
Concentration of Cu in plant organs of *Rhazya stricta* grown in soil treated with 10, 50 and 100 mg Cu/kg soil in an open greenhouse. Different lowercase letters indicate significant differences between plant parts for each soil metal concentration, and different capital letters indicate a significant difference between soil metal concentrations for each plant part (*p* < 0.05).

The concentration of Cu by *R. stricta* depended on the plant organ and the treatment level (Figure 5). The Cu concentration in the roots increased with the increasing level of Cu in the soil and reached 50.10 mg/kg in the 100 mg/kg Cu soil treatment. In both the 50 and 100 mg/kg treatments, the Cu concentration in leaves was higher than that in the stem, while the bioaccumulation value in the stem was higher than that in leaves for plants grown in soil treated with 10 mg/kg Cu.

**Figure 6:**
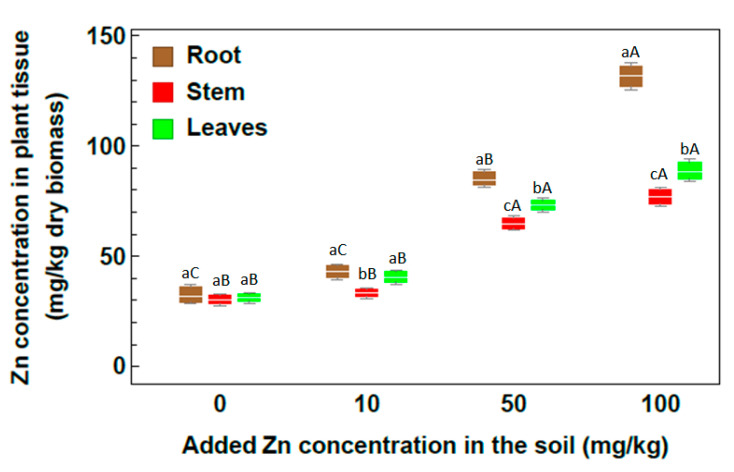
Concentration of Zn in plant organs of *Rhazya stricta* grown in soil treated with 10, 50 and 100 mg Zn/kg soil in an open greenhouse. Different lowercase letters indicate significant differences between plant parts for each soil metal concentration, and different capital letters indicate a significant difference between soil metal concentrations for each plant part (*p* < 0.05).

Considering the concentration of Zn in *R. stricta* (Figure 6), Zn bioaccumulation increased with increasing Zn content in the soil. By comparing the Zn concentrations for all considered treatments, the highest Zn concentration values were observed at 100 mg Zn/kg. For 100 mg Zn/kg, the highest Zn concentration was recorded in roots with value 131.66 mg/kg, followed by leaves with value 88.98 mg/kg. This indicates that *Rhazya stricta* plants were successful in absorbing Zn heavy metal and transferring it to the aerial plant parts. Generally, for all considered treatments, *R. stricta* plants showed high efficiency in Zn heavy metal uptake and accumulation.

Regarding the element distribution in plant organs, the concentrations of both essential and nonessential elements were significantly higher in root than in shoot, and essential elements such as Zn and Cu were efficiently trapped in roots; however, both root and shoot are considered as the main sites of metal accumulation.

### 2.3. Correlation Analysis

Correlation coefficients between heavy metal concentrations in soil and heavy metal concentrations in *Rhazya stricta* plant organs based on all treatments are listed in Table 1.

For Zn treatments, significant positive correlation between Zn concentrations in soil and Zn concentrations in root, stem and leaf (*r* = 0.996, 0.968, and 0.964, respectively) were observed. For Pb and Cu, a significant positive correlation was detected for root only with values (*r* = 0.988 and 0.95, respectively). Under different Cd treatments, negative correlations were detected (*r* = −0.25, −0.20) for stem and leaf, respectively.

### 2.4. Bioconcentration and Translocation Factors

**Figure 7:**
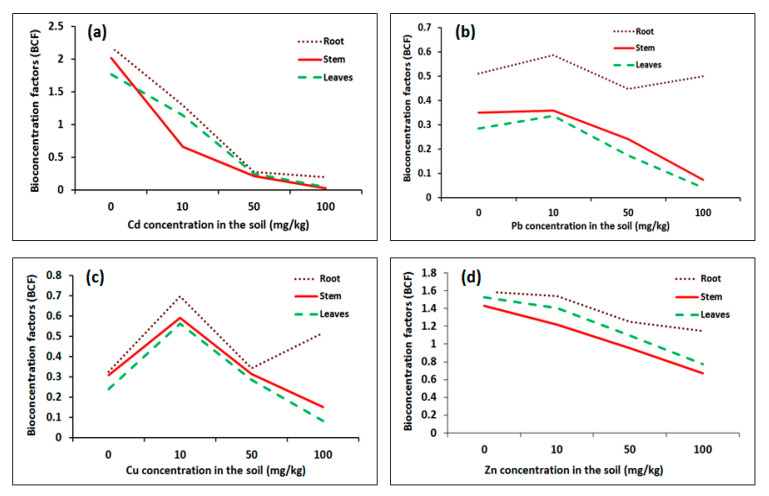
Bioconcentration factor (BCF) of *Rhazya stricta* plant organs grown in soil treated with heavy metal treatments of 10, 50 and 100 mg/kg soil in an open greenhouse. (**a**) BCF of *Rhazya stricta* plant organs in soil treated with Cd, (**b**) BCF of *Rhazya stricta* plant organs in soil treated with Pb, (**c**) BCF of *Rhazya stricta* plant organs in soil treated with Cu, (**d**) BCF of *Rhazya stricta* plant organs in soil treated with Zn.

The calculated bioconcentration factor (BCF) demonstrated that plants grown under the 10 mg/kg Cd soil treatment had high BCF values (Figure 7a); the BCF values in roots and leaves exceeded unity and were 1.30 and 1.14, respectively. In contrast, plants grown in soil Cd treatments of 50 and 100 mg/kg had a lower ability to concentrate Cd in their organs.

The BCF of Pb Figure 7b had higher values for 10 mg/kg Pb soil treatment and reached 0.57, 0.36 and 0.34 in roots, stems and leaves, respectively. For the 100 mg/kg Pb treatment, the BCF in plant roots was 0.5.

For Cu, the BCF values for different plant organs under 10 mg/kg were higher than the BCF values recorded in the other treatments (Figure 7c), whereas for 50 mg/kg Cu treatment, the BCF values were reduced by 50% compared to the results of 10 mg/kg. The plant roots showed BCF values of 0.52 under 100 mg/kg Cu treatment, which was higher than the values for stems and leaves.

The plants grown in different soil Zn treatments (Figure 7d) showed BCF values of more than one for plants grown in the 10, mg/kg and 50 mg/gr Zn treatments. The BCF values for 10 mg/kg Zn treatments were 1.54, 1.22 and 1.41 in the roots, stems and leaves, respectively, and for 50 mg/kg, the BCF values were 1.25, 1.02 and 1.1 in the roots, stems and leaves, respectively.

For all treatments, the highest BCF value was found in the root at 10 mg/kg Zn metal treatment, which was 1.538, followed by those of Cd, Cu and Pb, with values of 1.30, 0.70 and 0.58, respectively. For stems, the BCF of Zn reached 1.22 under 10 mg/kg treatment, followed by those of Cd, Cu and Pb with values of 0.66, 0.59 and 0.36, respectively. In the case of plant leaves, the same trend as stem was observed. The order of heavy metals uptake from soil by *R. stricta* from higher to lower values was Zn > Cd > Cu > Pb.

The translocation factor (TF) of the different heavy metals was less than unity. High TF values were recorded in the control, 10 mg/kg and 50 mg/kg soil treatments. In contrast, low TF values were measured in plants grown under 100 mg/kg treatment (Table 2). Generally, *R. stricta* plants show high metal bioaccumulation and translocation from roots to stems and leaves.

## 3. Discussion

The use of vegetation and associated microflora is becoming an important green technology for the in situ remediation of soils polluted with different heavy metals without the need of other chemical or artificial techniques, which have proved to be costly and ineffective for remediation at a large scale [5,23,35]. This is due to the role of vegetation, particularly of keystone species, in conserving the soil from erosion and preventing the leaching of pollutants to the underground water and the surrounding ecosystems.

For morphological features, the obtained results indicated the ability of *R. stricta* plants to grow in heavy metal-contaminated soil without significant morphological changes. This might be due to the ability of these plants to remove the heavy metals from contaminated soil without remarkable effect on their growth. These results illustrated that *R. stricta* plants can accumulate heavy metals while having normal morphological appearance. Therefore, they should be checked for contaminant load before processing them for clinical purposes. This suggestion is consistent with the suggestion introduced by Shah et al. [34] who illustrated that some medicinal plants, including *R. stricta*, should be checked for contaminant load before processing them for pharmaceutical purposes or for local human consumption. The non-significant root growth observed in this study might be due to the root confinement within a limited volume of the minipots with ample and regular water irrigation for the plants grown in the greenhouse [36].

The results for Cd heavy metal clarify that 50 mg/kg treatments showed the highest efficiency for bioaccumulation of heavy metal by root and transferred it to the aerial parts. Studies on lettuce plants by [37] on the bioaccumulation of Cd showed similar results to those obtained for *R. stricta*. Lettuce plants represented a good example for Cd absorption from the soil by root, subsequently translocated via xylem vessels to leaves.

Lead is naturally presented in in the earth’s crust at concentrations of 8–20 mg/kg soil [38]. The phytoremediation mechanism would be suggested to remediate the elevated lead concentrations from contaminated soil. The study of [39] demonstrated the ability of *Cynodon dactylon* to accumulate Pb, where the shoot-root ratio, which helps to verify the translocation of heavy metals through the plant, showed that the amount of accumulated Pb was higher in roots and subterranean rhizomes than in aerial shoots. The same results were obtained with *R. stricta*; the plants showed a high ability to accumulate Pb within the roots even at a high heavy metal concentration of 100 mg/kg in the soil. The Pb content in the plant roots increased with the increased Pb levels in soil, as the dependence of the Pb content on soil characteristics may control heavy metal availability for plants [39,40].

The normal concentration range of Cu in plants is from 5–20 mg/kg [41]. Obtained results for Cu concentration illustrate that by increasing the level of Cu in soil, an increase in Cu concentration occurs in the root. Similar results were obtained by Ibrahim et al. [32], who reported that the amount of Cu accumulated in the roots of *Lycium shawii*, *Phragmites australis*, and *R. stricta* reached 2 to 4.5 times that accumulated in the shoot. The results of this study showed also that leaves of *R. stricta* accumulate Cu more than stem in both 50 and 100 mg/kg treatments. These results agree with the results given in [42], which stated that the shoot of *Convolvulus arvensis* accumulated Cu to more than 560 mg/kg dry plant tissue in heavy metal-contaminated sites. The same was demonstrated by Khattak et al. [33], who found that the highest Cu concentration was detected within *R. stricta* plants compared to *C. colocynthis* and *E. cornigera* plants.

Zinc is a micronutrient element that helps in the formation of a large number of different enzymes and plays an important role in regulatory and catalytic functions. Zn has a significant role in pollen formation, DNA synthesis and also enhances the antioxidant enzymes and photosynthesis within plant tissues [43]. Zn concentrations in the unpolluted soil depend on geology and specific site conditions. Zn toxic effects occur at concentrations greater than 100 mg/kg soil as illustrated by Mertens and Smolders [44]. To discuss low or moderate pollution levels, the concentrations 10, 50 and 100 mg/kg of Zn heavy metal were added to soil. Zn hyperaccumulator plants accumulate more than 10 mg/g dry weight in the aerial parts when growing in their natural environment [45]. Results obtained by [46] for *Thlaspi caerulescens* plants, reported high Zn accumulation in plant tissues in soil treated with up to 100 mg/kg soil. The same results were obtained in the present study, since it was clear that *R. stricta* plants showed high efficiency to absorb Zn heavy metal and transfer it to the aerial parts with all treatments. In spite of the harmful effect of zinc heavy metal on plants, it is less toxic compared to several other heavy metals and is relatively harmless [47,48]. So it was clear that with all treatments of Zn concentration, *R. stricta* plants showed high efficiency for absorbing Zn heavy metal and transferring it to the aerial parts.

By increasing heavy metal concentrations in the soil, the rate of heavy metal uptake by the root, and the bioaccumulation as well as the translocation in the plant, increased. This increase exceeds the normal levels of these metals in plants. Such results are in agreement with the findings of [49,50] in other species that demonstrated their ability to remediate rhizosphere soils due to the high uptake and bioaccumulation of heavy metals in their tissues.

In this study, within all treatments of Zn, Cu, Pb, and Cd metals, root showed the highest metal concentrations. Similar results were illustrated by Badr et al. [30], who reported that after testing several native plants, including the *R. stricta* plant, for polluted metal phytoextraction, all plants accumulated high concentrations of toxic pollutants within their root.

In this study, at 100 mg/kg Cd, Pb, and Cu heavy metals concentration, a significant increase in heavy metals concentration was remarked in the root, whereas a significant decrease was remarked for stem and leaves. This might be due to the high concentration of heavy metals in the root, which caused severe physiological damage the root of the plant and reduced its ability to translocate heavy metals to aerial parts of the plant. Therefore, a significant decrease in heavy metal concentrations was remarked for stem and leaves at 100 mg/kg soil treatment [51]. Al-qahtani [31] studied the efficiency of seven native plants, including the *R. stricta* plant, for bioaccumulation of eight heavy metals. The results showed that all selected plants had efficiency in accumulating and translocating several heavy metals including Cd, Pb, Cu, and Zn, with different levels. The *P. australis* and *C. laevigatus* plants showed higher capability for heavy metals accumulation than other studied native plants including *R. stricta*.

Bioconcentration factor (BCF) and translocation factor (TF) are parameters that can be used to estimate plant phytoremediation potential [52]. Based on the metal availability in soil and its uptake by plants, the bioaccumulation and translocation factor values indicate the ability of plants to accumulate and transfer heavy metals within their tissues [53,54,55,56]. The translocation of the accumulated heavy metals from the roots to the shoots starts after the roots lose their ability to phytostabilize or to store the heavy metals [57]. The values of bioconcentration factor (BCF) given by Al-qahtani [31] were higher than the values recorded in this study; this may be due to the fact that the Al-qahtani [31] study was done in the field with mature plants, while our experiment was done in greenhouse for plants germinated only for three months.

The efficiency of heavy metal translocation from root to shoot can be detected according to TF values [58]. Effective heavy metal transfer was suggested at TF values greater than one, whereas at TF values of less than one, these types of plants could accumulate metals in roots and rhizomes more than in shoots or leaves, which suggested ineffective metal transfer [55].

In this study, the results for *R. stricta* demonstrated that the bioconcentration factor (BCF) of Zn and Cd heavy metals in root and shoot indicates the plant’s capacity to accumulate these metals. The translocation factor (TF) values of the four considered heavy metals, however, were near to one, which suggests that the plant remediates heavy metals by phytostabilization through concentrating the metals in the root. Both Zn and Cu showed the highest (TF) values. The same pattern for translocation factor of *R. stricta* was obtained by Al-Farraj et al. [27] who demonstrated that Zinc heavy metal attained the highest values of translocation factor (TF) compared to Cd, Cu, and Pb heavy metals. Phytostabilization of heavy metals limits its transport as well as keeping animals from toxic species ingestion, and consequently prevents transmission across the food chain.

## 4. Materials and Methods

### 4.1. Study Species

*Rhazya stricta* Decne. (Apocynaceae) is a native evergreen perennial herb that is widely distributed in Saudi Arabia. Plant populations and communities dominated by *R. stricta* are found in depressions and overgrazed sandy flats with silty and sandy soils [24]. As an unpalatable species for grazing domestic animals, the species dominates overgrazed or deteriorated grazing lands and shapes the physiognomy of the landscape as a keystone species. Mature seeds were collected from plant populations growing naturally in sand flats along Taif and Makkah roads, Saudi Arabia and used in pot experiments. Filled seeds were collected at the fruit dehiscence/seed dispersal stage. The seeds were collected from 50 individual plants selected randomly in one population covering approximately 2 km^2^ of land area.

### 4.2. Pot Experiment

*R. stricta* seeds were germinated and grown for three weeks in untreated soil in minipot seedling trays. The three-week-old plants were transplanted into plastic pots of 22 cm in diameter and 25 cm depth. Healthy plants of similar height were selected for transplantation. The pots contained soil collected from the plants’ natural habitat. The soil was sandy-loamy with a slightly alkaline pH 7.3, a low organic matter content (0.48%), a total carbonate concentration of 19.3%, and electric conductivity of 986 mhos/cm. The concentrations of the different heavy metals in the natural (control) soil were Cd 3.2 mg/kg soil, Pb 8.1 mg/kg soil, Cu 17.1 mg/kg, and Zn 20.5 mg/kg.

The experiment was conducted in an open greenhouse starting in mid-February and terminating by the end of May. During the experimental period, the average maximum temperature was 28 °C, ranging from 22 °C to 32 °C, and the average minimum temperature was 15 °C, ranging from 14 °C to 18 °C. Plants were watered regularly to the maximum 100% field capacity, which required watering every 2–3 days.

For soil treatments, the pots were divided into groups. Five pots were considered with individual plants, as five replicates for each group. The first group was for the control without heavy metals. The other groups were for Cd, Pb, Cu, and Zn treatments as five replicates for each considered concentration. For the heavy metal treatments, Cd was applied as CdCl_2_·2 ½ H_2_O, Pb was applied as Pb (NO_3_)_2_, Cu was applied as CuSO_4_·5 H_2_O, and Zn was applied as ZnSO_4_·7 H_2_O.

To overcome the potential stress of heavy metal treatments at the seedling stage of plant growth, *R. stricta* seeds were germinated and grown for three weeks [59,60] in untreated soil in minipot seedling trays. The three-week-old plants were transplanted into plastic pots of 22 cm in diameter and 25 cm depth. Since high concentrations of toxic heavy metals affected seedling germination [61,62,63,64], selected heavy metals were mixed in the soil at rates of 10, 50, and 100 mg/kg soil [63,65,66]. The considered heavy metal concentrations are also appropriate for the pollution range in the considered area.

After the termination of the experiments, the individual plants in each pot were harvested and separated into their corresponding organs: roots, stems and leaves. The separated plant materials were washed with tap water followed by distilled water to remove dust and soil particles. The material was oven dried at 80 °C for 48 h to a constant weight, ground into a fine powder and kept dry in paper bags in a desiccator for subsequent heavy metal analysis.

### 4.3. Growth Rate

For fresh and dry weight of *R. stricta* plants, three month grown plants at different heavy metal concentrations were harvested. The plants were washed with tap water followed by distilled water to determine the total fresh weight, and then plants were oven dried at 80 °C for 48 h to a constant weight to detect the total dry weight. Dried samples were separated into their corresponding organs: roots, stems and leaves, then percentages of resources allocated across different organs were calculated.

### 4.4. Heavy Metal Analysis

The heavy metal content was determined in 0.5 g plant powder after digestion in 65% concentrated HNO_3_ and 2 mL HClO_4_ following the procedure described by [67]. The heavy metal concentration was measured spectrophotometrically by atomic absorption (Thermo Scientific model ice 3000 SERIES AA) under the specific conditions shown in Table 3.

### 4.5. Calculation of Bioconcentration and Translocation Factors

The bioconcentration factor (BCF), a dimensionless factor, was calculated as the ratio of a given metal concentration in the plant tissues at harvest (mg/kg dry weight) to the concentration of the metal in the soil medium according to the following equation [68].

$$BCF\;=\;\;\frac{C_{harvested\;tissue}}{C_{soil}}$$

where *C_harvested tissue_* is the metal concentration in the plant tissues (roots, stems or leaves) and *C_soil_* is the metal concentration in the soil.

Some plants showed an ability to translocate absorbed heavy metals from the root to the shoot system, and the ratio of the concentration of a heavy metal in plant shoots and its concentration in roots is defined as the translocation factor (*TF*).

The translocation factor (*TF*) was calculated as (shoot concentration/root concentration) mg/kg dry weight [69].

$$TF\;=\;\;\frac{C_{Shoot\;}}{C_{root}}$$

where *C_shoot_* is the metal concentration in the plant tissues (stems or leaves) and *C_root_* is the metal concentration in the plant root. The *TF* is a dimensionless factor; a higher ratio implies higher translocation capability.

### 4.6. Statistical Analysis

Each experiment was conducted with five replicates. The results are shown as the mean ± standard deviation. The experimental data were analyzed using the SPSS-22 statistical software package for PC. The heavy metal concentrations in the control and experimental treatments were compared using Student’s *t* test. To test the difference in heavy metal concentrations between different samples, the data were checked for normality and homogeneity and analyzed by two-way ANOVA using Fisher’s post hoc test comparison method. To check the data normality and homogeneity, Levene’s test of equality of error variances was applied to test the homogeneity and the Kolmogorov–Smirnov test was used to test the normality. The differences between samples were considered statistically significant at *p* < 0.05. The correlation coefficient (*r*) of the various metals with the concentration in the plants was calculated using Pearson’s regression equation between metal concentration in soil and metal concentration in respective plant organ based on all treatments for the particular metal [5,22,23].

## 5. Conclusions

The heavy metal concentration in the roots of *R. stricta* was higher than that in stems and leaves under the tested Cd, Pb, Cu and Zn soil treatments. This may be due to the increase in the heavy metal concentration in the soil leading to an increase in the bioaccumulation in roots. The phytoremediation ability of *R. stricta* prevents the mobility of the heavy metals in the soil to the surrounding areas, which suggests that this keystone species is a potential phytoremediator for lightly polluted soils. The bioaccumulation of heavy metals by *R. stricta*, including its ability to absorb and accumulate heavy metals, indicates that this species is a candidate for large-scale in situ phytoremediation of lightly polluted soils in arid regions. Further field studies are required to assess the phytoextraction, phytostabilization and rhizofiltration behavior of this species in natural habitats, as these are useful strategies in the remediation of heavy metal-polluted soils in plant communities in arid lands.

## Figures and Tables

**Table 1:** Correlation coefficients (*r*) between heavy metal concentrations in the soil and heavy metal concentrations in the plant organs.

Metal	Root	Stem	Leaf
Cd	0.856	−0.250	−0.201
Pb	0.988 *	0.478	0.210
Cu	0.95 *	0.576	0.117
Zn	0.996 *	0.968 *	0.964 *

* Correlations are significant at the 0.05 level (*n* = 5).

**Table 2:** Translocation factors (TF) of Cd, Pb, Cu and Zn in *R. stricta* plants grown in soils treated with heavy metal concentrations of 10, 50 and 100 mg/kg and natural soil as a control.

Metal Conc. in Soil	Translocation Factor in Plant Shoots							
	Cd		Pb		Cu		Zn	
	TF Stem	TF Leaves	TF Stem	TF Leaves	TF Stem	TF Leaves	TF Stem	TF Leaves
0	0.92 ^a^	0.81 ^a^	0.69 ^a^	0.56 ^a^	0.74 ^a^	0.95 ^a^	0.90 ^a^	0.96 ^a^
10	0.51 ^b^	0.88 ^a^	0.61 ^a^	0.57 ^a^	0.81 ^a^	0.35 ^b^	0.79 ^b^	0.92 ^a^
50	0.79 ^a^	0.91 ^a^	0.74 ^a^	0.53 ^b^	0.83 ^a^	0.92 ^a^	0.76 ^b^	0.88 ^a^
100	0.13 ^c^	0.23 ^b^	0.15 ^b^	0.08 ^c^	0.16 ^b^	0.29 ^b^	0.59 ^c^	0.68 ^b^
L.S.D	0.15	0.21	0.12	0.02	0.27	0.12	0.01	0.12

The different letters refer to significant differences at *p* < 0.05.

**Table 3:** Conditions for the measurement of heavy metal concentrations by atomic absorption.

Heavy Metal	Lamp Current (mA)	Fuel	Support	Wavelength (nm)
Cadmium	5	Acetylene	Air	228.8
Lead	10	Acetylene	Air	217
Copper	5	Acetylene	Air	324.8
Zinc	10	Acetylene	Air	213.9
